# Omega-3 Polyunsaturated Fatty Acids in Managing Comorbid Mood Disorders in Chronic Obstructive Pulmonary Disease (COPD): A Review

**DOI:** 10.3390/jcm12072653

**Published:** 2023-04-02

**Authors:** Halliru Zailani, Senthil Kumaran Satyanarayanan, Wei-Chih Liao, Hsien-Feng Liao, Shih-Yi Huang, Piotr Gałecki, Kuan-Pin Su, Jane Pei-Chen Chang

**Affiliations:** 1 Mind-Body Interface Laboratory (MBI-Lab), Department of Psychiatry, China Medical University Hospital, Taichung 404, Taiwan; halliruzyln55@gmail.com (H.Z.);; 2 Graduate Institute of Nutrition, China Medical University, Taichung 404, Taiwan; 3 Division of Pulmonary and Critical Medicine, Department of Internal Medicine, China Medical University Hospital, Taichung 404, Taiwan; 4 School of Nutrition and Health Sciences, Taipei Medical University, Taipei 110, Taiwan; 5 Nutrition Research Centre, Taipei Medical University Hospital, Taipei 110, Taiwan; 6 Department of Adult Psychiatry, Medical University of Lodz, 91-229 Lodz, Poland; 7 College of Medicine, China Medical University, Taichung 404, Taiwan; 8 Graduate Institute of Biomedical Sciences, China Medical University, Taichung 404, Taiwan; 9 An-Nan Hospital, China Medical University, Tainan 833, Taiwan

**Keywords:** chronic obstructive pulmonary disease, anxiety, depression, oxidative stress, inflammation, omega-3 polyunsaturated fatty acids

## Abstract

Chronic obstructive pulmonary disease (COPD) is the third-leading cause of mortality globally, significantly affecting people over 40 years old. COPD is often comorbid with mood disorders; however, they are frequently neglected or undiagnosed in COPD management, thus resulting in unintended treatment outcomes and higher mortality associated with the disease. Although the exact link between COPD and mood disorders remains to be ascertained, there is a broader opinion that inflammatory reactions in the lungs, blood, and inflammation-induced changes in the brain could orchestrate the onset of mood disorders in COPD. Although the current management of mood disorders such as depression in COPD involves using antidepressants, their use has been limited due to tolerability issues. On the other hand, as omega-3 polyunsaturated fatty acids (n-3 PUFAs) play a vital role in regulating inflammatory responses, they could be promising alternatives in managing mood disorders in COPD. This review discusses comorbid mood disorders in COPD as well as their influence on the progression and management of COPD. The underlying mechanisms of comorbid mood disorders in COPD will also be discussed, along with the potential role of n-3 PUFAs in managing these conditions.

## 1. Introduction

### 1.1. Chronic Obstructive Pulmonary Disease

Chronic obstructive pulmonary disease (COPD) is an age- and lifestyle-dependent respiratory disease characterized by high morbidity and mortality. In 2019, COPD was considered the third-leading cause of mortality worldwide (about 3.23 million deaths) after ischemic heart disease and stroke, and about 80% of these deaths were recorded in low-and middle-income countries [1]. Symptoms of COPD include cough (frequently with phlegm), dyspnea, and lethargy. COPD can be caused by either hereditary or environmental factors [2]. Hereditary impairment of the serine protease inhibitor (serpin) enzyme, Alpha-1 antitrypsin (AAT), increases susceptibility to COPD [3]. On the other hand, long-term cigarette smoking (CS) and exposure to dust and environmental contaminants are lifestyle causative factors of COPD [4]. While many causes and risk factors are associated with COPD, CS has been shown to have the strongest association [5].

**Figure 1:**
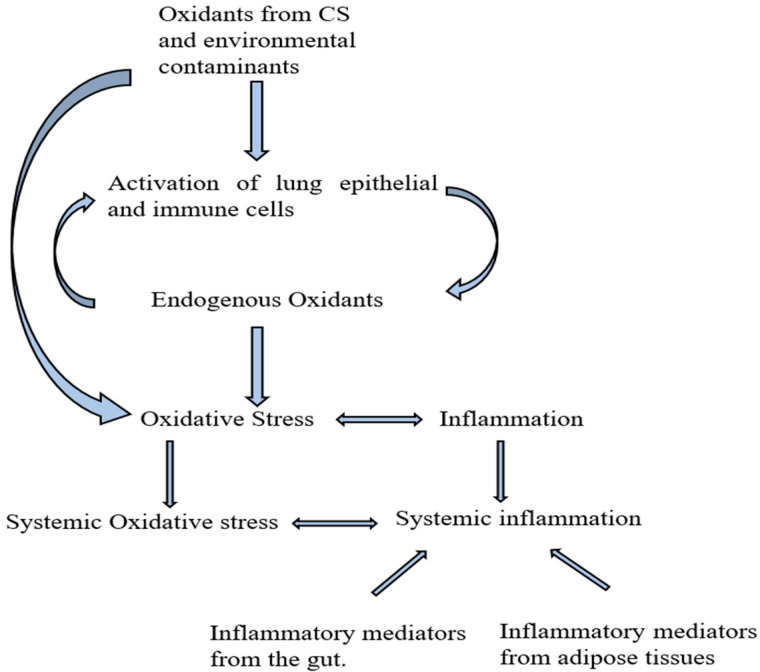
Sources of inflammatory mediators in COPD. CS: cigarette smoking. Oxidants from cigarette smoking and other environmental contaminants activate the lung epithelial and immune cells, leading to more oxidants from the immune cells, resulting in local oxidative stress. Oxidative stress initiates a series of activities that ultimately lead to local inflammation. The accumulated pro-inflammatory mediators in the lungs can leak into the blood, causing systemic inflammation. Pro-inflammatory mediators in COPD can also originate from the gut and adipose tissues.

COPD is characterized by oxidative stress, the systemic imbalance between the reactive oxygen species (ROS) and the body’s antioxidant defense system [6], and inflammation [7,8]. ROS produced from long-term CS or exposure to environmental pollutants accumulates in the lungs, leading to a cascade of immune reactions that produce pro-inflammatory mediators resulting in local inflammation (Figure 1). ROS in the lungs leads to the activation of epithelial and immune cells (neutrophils and macrophages), which produces more oxidant species and inflammatory mediators, thereby creating local OS and inflammation. ROS and inflammatory mediators can spill over to the systemic circulation to create systemic oxidative stress and inflammation. Systemic inflammation associated with COPD can also originate from the leakages of inflammatory mediators from adipose tissues [9] and the gut [10,11]. Obesity is common in the early stages of COPD [12,13,14], and adipose tissues produce significant pro-inflammatory mediators in obesity [9,15]. Another potential source of inflammatory markers in COPD is the gut, partly due to inflammatory bowel disease (IBD), a common comorbidity of COPD [10,11]. COPD patients have been reported to have increased colon and small intestine permeability [16], also commonly seen in IBD [17], contributing to gut inflammation, and may facilitate pro-inflammatory cytokine leakage. Systemic inflammation is widely believed to play a crucial role in developing COPD-related comorbidities.

Extrapulmonary comorbid mood disorders often characterize COPD and are believed to result from a manifestation of inflammation associated with COPD. Anxiety and depression are the most common mood disorders associated with COPD; however, they often go unrecognized or undiagnosed in treating COPD [18]. Comorbid anxiety and depression may reduce adherence to the COPD treatment protocols and poor clinic attendance, contribute to poorer quality of life (QOL), and increase the recurrence of exacerbation and mortality among COPD patients [18,19]. As a result, a special emphasis on detecting and controlling neuropsychiatric comorbidities in the clinical care of COPD might help minimize the COPD burden and significantly improve the QOL of the patients [20]. Comorbid anxiety and depression in COPD are commonly managed with pharmacological drugs. However, adherence to these drugs has been hindered by stigma toward mental diseases, general misconceptions about the drugs, and their potential undesirable adverse effects [21,22,23]. Given the importance of managing mood disorders in managing COPD, there is a need for a safe alternative prophylactic therapy with reduced potential adverse effects in managing mood disorders in COPD. Interestingly, omega-3 polyunsaturated fatty acids (n-3 PUFAs) may have the potential to help manage such inflammation-driven conditions with more tolerable side effects.

### 1.2. Polyunsaturated Fatty Acids (PUFAs)

**Figure 2:**
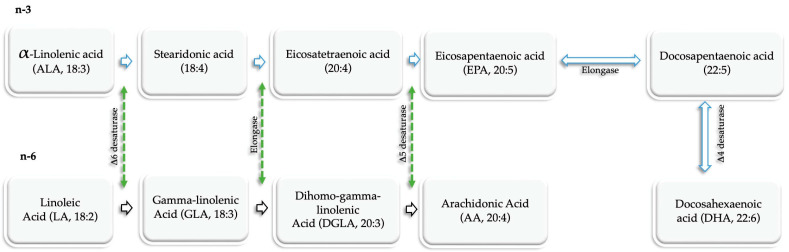
De novo synthesis of n-3 and n-6 PUFAs. n-3 PUFAs are derived from ALA. ALA in the liver is desaturated to stearidonic acid and then elongated to eicosatetraenoic acid, which is then desaturated to EPA. DHA is synthesized from EPA in a two-step reaction sequentially catalyzed by elongase and desaturase. The biosynthesis of n-6 PUFAs starts with the conversion of linoleic acid to DGLA via desaturation and elongation reactions. DGLA is then further desaturated to arachidonic acid. AA: Arachidonic Acid, ALA: α-linolenic acid, DHA: docosahexaenoic acid, DGLA: dihomo-gamma-linoleic acid, EPA: eicosapentaenoic acid, GLA: gamma-linolenic acid, LA: linoleic acid, n-3: omega-3 polyunsaturated fatty acids, n-6: omega-6 polyunsaturated fatty acids, PUFAs: polyunsaturated fatty acids.

PUFAs are a special group of fatty acids characterized by the presence of two or more double bonds. PUFAs can be either n-3 or omega-6 (n-6) depending on the position of the first double bond when counting from the methyl end of the fatty acid chain. N n-3 and n-6 PUFAs have their first double bond on the C-3 and C-6 positions when counting from the methyl end. PUFAs are essential fatty acids physiologically found as components of cell membranes. Because the body cannot synthesize them, PUFAs are normally obtained exogenously from dietary sources [24]. α-linolenic acid (ALA) is the simplest of n-3 PUFAs in vegetable oil such as flaxseed, canola, and soybean. ALA can be metabolized in the liver by elongation and/or desaturation processes catalyzed by enzymes to generate the more essential and physiologically active long-chain n-3 PUFAs, eicosapentaenoic acid (EPA), and docosahexaenoic acid (DHA). EPA comprises 20 carbon atoms and five double bonds (20:5n-3), while DHA comprises 22 carbon atoms and six double bonds (22:6n-3). Dietary sources of n-3 PUFAs include deep-sea fishes (Table 1). Linoleic acid is the simplest n-6 PUFA chiefly found in corn, safflower, and sunflower and serves as the substrate for synthesizing other n-6 PUFAs, including arachidonic acid (AA), in the body (Figure 2). The conversion rate of ALA to EPA and DHA is very limited and cannot meet the body’s physiological requirement; hence, the need arises to obtain them from dietary sources such as deep-sea fish and fish oils. While 3 PUFAs are anti-inflammatory, n-6 PUFAs are pro-inflammatory [25,26]. Indeed, n-3 PUFAs inhibit the production of several pro-inflammatory cytokines as well as resolve inflammation through their metabolites called specialized pro-resolving mediators (SPMs), which include resolvin D (RvD) and E (RvE) series, maresins (MaR) and protectins (PD) [27,28]. On the other hand, AA has been proven to trigger inflammation as it serves as the precursor for synthesizing pro-inflammatory eicosanoids, such as prostaglandins E2 [26]. Consequently, diets high in n-3 PUFAs and low in n-6 PUFAs may reduce the occurrence of diseases driven by inflammation. Moreover, studies have reported associations between the inadequate intake or reduced plasma levels of n-3 PUFAs and the development of inflammatory diseases, such as mood disorders [29,30,31]. There is insufficient information on the amount of daily n-3 PUFAs intake required to achieve an anti-inflammatory response. However, it is hinted that a minimum daily intake of 2 g n-3 PUFAs is required to manifest their anti-inflammatory effects [32].

To our knowledge, there is currently no review of the potential of n-3 PUFAs in managing comorbid anxiety and depression in COPD. Therefore, in this review, we aimed to discuss comorbid anxiety and depression in COPD, and their influence on the progression and management of the disease. Furthermore, we will discuss the underlying mechanisms, including epigenetics, associated with comorbid mood disorders in COPD. Finally, we will discuss the potential roles of n-3 PUFAs in managing COPD comorbid mood disorders.

## 2. Comorbid Conditions of Mood Disorders in COPD

Evidence has shown that anxiety and depression are common in COPD. COPD patients have a greater prevalence of depression and anxiety than unaffected persons [33] and are reported to have a higher relative risk of depression [34]. A large cross-sectional study (n = 4803) reported a higher rate of depression in patients with COPD (62.1%) than those with other chronic diseases [35]. In addition, a large multicenter cohort study reported a higher rate of depression in COPD patients (26%) compared with COPD-free smokers (12%) and healthy non-smokers (7%) [36]. Depression and anxiety correlate with COPD severity [37,38]. The rate of depression in stable COPD individuals in outpatient clinics ranges from 10% to 57% [37,39,40,41], while anxiety prevalence ranges from 7% to 50% [39,42]. A meta-analysis reported a higher rate of depression among COPD patients (27.1%) compared with controls (10.0%) [43]. The growing rate of anxiety and depression in COPD patients is detrimental and a significant barrier to COPD management since psychiatric conditions may impede COPD treatment adherence [44]. Comorbid anxiety and depression lead to increased mortality and poor outcomes in COPD management [41,45,46]. Similarly, COPD patients suffering from anxiety or depression have a higher incidence of hospitalizations due to COPD exacerbations and a lower QOL [46,47]. Similarly, worsening dyspnea perception in anxious and depressed individuals may lead to increased hospitalizations owing to exacerbation [48,49].

The global initiative for chronic obstructive lung disease (GOLD) 2020 recommends that anxiety and depression be treated independently of COPD treatment [4]. As a result, anxiety and depression in COPD are currently managed with the use of antidepressants. However, the efficacy of antidepressants in COPD remains to be concluded as the trials were limited by small sample sizes resulting from high dropout rates, sample heterogeneity, and variability in the depression rating and monitoring scales [50]. COPD patients often reject antidepressants due to misconceptions about depression and the side effects associated with the antidepressants [50,51]. Some side effects include but are not limited to suicidal ideation, blurred vision, nausea and vomiting, insomnia, dizziness, worsening anxiety, constipation etc. [50]. In addition, a recent retrospective secondary study reported a small but significant association between the use of serotonergic antidepressants and a higher mortality rate and lung-related diseases among older adult COPD patients [52]. In sum, poor compliance with antidepressants in COPD necessitates the development of alternative therapy with minimal side effects.

## 3. Underlying Mechanisms Associated with Mood Disorders in COPD

Inflammation characterizes anxiety and depression [53,54,55,56,57]. Evidence has shown that patients with depression exhibit all essential signs of inflammation, such as high levels of chemokines, cytokine receptors, and pro-inflammatory cytokines [55,58]. Furthermore, innate immune response activation and cytokine release are linked to the development of mental and cognitive disorders [59,60,61]. Similarly, when pro-inflammatory cytokines or stimulators are administered to people who are not depressed, depressive symptoms arise [55,62]. Inflammation is similarly indicated in the development and progression of COPD [63] (Table 2). Indeed, earlier studies reported increased plasma inflammatory markers such as leukocytes, C-Reactive Protein (CRP), fibrinogen, interleukin (IL)-6, tumor necrosis factor (TNF)-α, and TNF-α receptor-1 in COPD patients compared to healthy individuals [64,65,66,67,68]. Moreover, a study that analyzed induced sputum samples from COPD patients (n = 26) and healthy controls (n = 21) reported significantly elevated levels of IL-6, IL-8, IL-13, and monocyte chemoattractant protein (MCP)-1 in the airway of COPD patients compared to healthy controls [69]. Elevated IL-6 levels in plasma are linked to depressive symptoms in COPD, independent of airflow limitation and comorbid risk factors for depression [70]. In addition, soluble TNF receptor-1 (sTNFR-1) was significantly associated with depression in COPD [65], while depression ratings have been positively associated with plasma TNF-α levels in COPD [71]. In addition, depression in COPD was linked with increased 24-h overall levels of sputum IL-1 and TNF-α and flattened diurnal salivary cortisol levels [72]. Moreover, compared with healthy controls, a higher level of IL-2, IL-6, and interferon (IFN)-γ was reported in patients with comorbid depression in COPD [73]. Moreover, IL-2 levels were significantly higher in the COPD group with comorbid depression [73].

Inflammation triggers depression through various mechanisms, including the upregulation of the serotonin transporter (SERT) gene in the brain [53], leading to accelerated serotonin reuptake and a decrease in extracellular levels [74,75]. Inflammatory cytokines can also increase the activity of indoleamine-2,3-dioxygenase (IDO)/tryptophan-2,3-dioxygenase (TDO) in the kynurenine (Kyn) pathway, resulting in increased degradation of tryptophan (TRP) to kynurenines, which reduces its plasma availability and transport to the brain [74,75]. Insufficient TRP in the brain results in a drastic reduction in brain serotonin synthesis, leading to depression. In COPD, increased activity of IDO, which was correlated with the disease severity, and a simultaneous reduction of blood TRP level were reported [76,77]. These findings suggest that inflammation leads to a cascade of events resulting in dysregulated TRP metabolism associated with anxiety and depression, highlighting the possible involvement of inflammation in the development of comorbid anxiety and depression in COPD.

**Figure 3:**
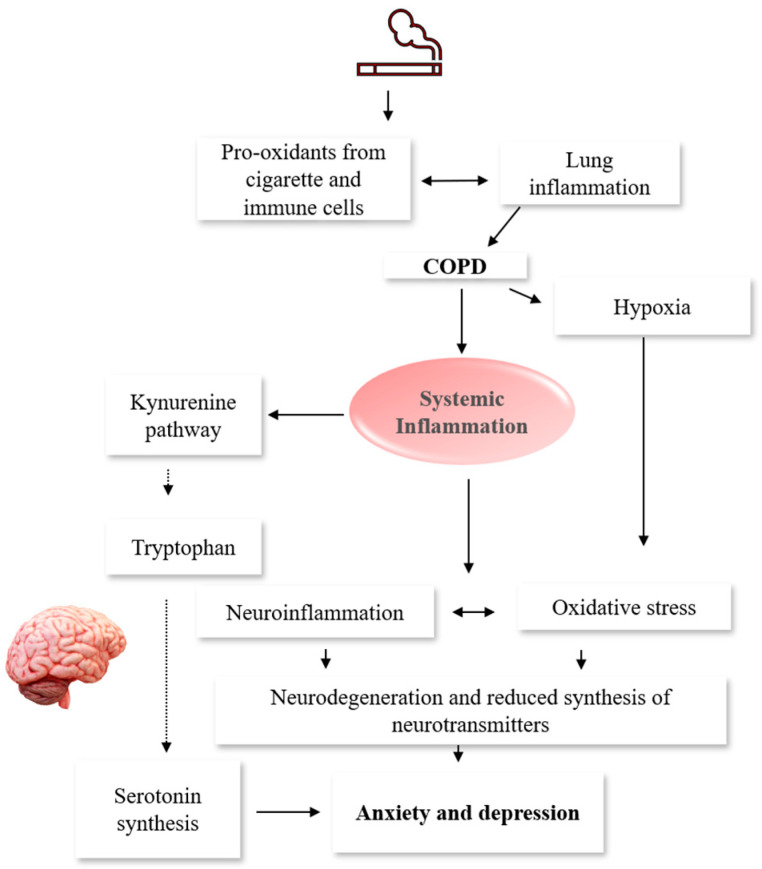
Putative Mechanisms of Increased Risk of COPD Patients to Mood Disorders. Reactive oxygen species from exposure to cigarette smoking accumulate in the lungs and trigger local inflammation. The lung inflammatory mediators spill into the plasma, where systemic inflammation sets in. Systemic inflammation activates the Kynurenine pathway. Hence, more tryptophan is degraded into kynurenines, limiting its transport into the brain for serotonin synthesis, leading to anxiety and depression. In addition, inflammatory mediators cross the blood–brain barrier, causing neurodegeneration and reduced synthesis of neurotransmitters, also resulting in anxiety and depression. Furthermore, hypoxia associated with COPD could lead to neuronal damage, thus interfering with the production of neurotransmitters. COPD: chronic obstructive pulmonary disease.

Given that COPD is associated with the degradation of alveolar capillaries and airflow restrictions, hypoxia is a logical consequence [78] (Figure 3). Hypoxia disrupts the synthesis of neurotransmitters [79], causing altered neuronal functions and ultimately cognitive deficit. Hypoxemia can enhance systemic inflammation, via the activation of the Nuclear Factor kappa-β (NF-kβ) [78], which regulates cellular inflammatory responses. Indeed, a positive correlation between hypoxemia following COPD exacerbations and systemic neutrophilic activity was reported [80]. Moreover, chronic hypoxia in mice increased circulating levels of IL-6 [81]. In addition to its systemic manifestation, chronic hypoxia exacerbates inflammatory responses in the brain, leading to various brain-related abnormalities. Indeed, previous studies have demonstrated that chronic intermittent hypoxia activates pro-inflammatory microglia phenotype (M1) [82,83,84]. This activation is often triggered by IFN-γ and lipopolysaccharide (LPS) and is associated with the production of several inflammatory cytokines and chemokines, including TNF-α, IL-6, IL-1β, IL-12, and CC chemokine ligand (CCL) 2 [85]. M1 microglia also expresses nicotinamide adenine dinucleotide phosphate (NADPH) oxidase and inducible nitric oxide synthase (iNOS), which produce reactive oxygen species (ROS) and nitric oxide (NO), respectively [85]. Increased levels of oxidative stress and pro-inflammatory cytokines in the hippocampus and cortex of mice [86] and rats [87] were reported following chronic hypoxia. In addition, elevated levels of pro-inflammatory cytokines and chemokines such as IL-6, TNF-α, chemokine C-C motif ligand 2 (CCL2), and CCL3 in both the hippocampus and cortex of hypoxic mice compared to normoxic mice were reported [83]. In sum, hypoxia may play a crucial role in the emergence of depression and other neurological disorders in the COPD population through its direct effect on the production of neurotransmitters, oxidative stress, or neuroinflammation via the activation of the M1.

Another possible mechanism underlying the development of anxiety and depression in COPD is oxidative stress (Table 2). Oxidative stress plays a crucial role in the pathogenesis of anxiety and depression [88,89,90]. Specifically, patients with depression have been reported to have higher levels of inducible nitric oxide synthase, superoxide dismutase, and nitrotyrosine in their plasma than healthy controls [91]. Additionally, individuals with anxiety and depression have been found to have elevated levels of 8-hydroxy-2′-deoxyguanosine (8-OHdG) in their plasma [92]. Moreover, a meta-analysis has shown that increased oxidative stress is associated with depression [93]. In COPD, CS and long-term exposure to air pollutants are the major causes of oxidative stress. A significant increase in the number of alveolar macrophages was noted in the lungs of individuals with COPD as compared to the healthy controls [94,95]. Moreover, these macrophages display higher activation levels, releasing an elevated quantity of superoxide anions and hydrogen peroxide [95]. In addition, activated neutrophils are increased in the lungs of patients with COPD, and activated peripheral blood neutrophils from COPD patients release a higher amount of ROS, which is critical during the acute exacerbations of COPD [95]. Oxidative stress in COPD could also be a function of overwhelmed endogenous antioxidant defense systems. The glutathione levels in the bronchoalveolar lavage fluid from COPD patients with frequent exacerbations are considerably lower than those of patients with stable COPD [96]. In addition, genetic polymorphisms associated with extracellular superoxide dismutase (SOD) as well as their expression in the sputum are frequently found in COPD patients [97,98]. Moreover, downregulation of nuclear factor erythroid 2-related factor 2 (Nrf2) and Nrf2-related, heme oxygenase-1, and glutamate-cysteine ligase catalytic subunit in the peripheral blood mononuclear cells, as well as elevated 8-isoprostane and decreased reduced glutathione levels were reported in patients with mild to moderate COPD compared with non-COPD controls [99]. Diminished levels of Nrf2 and another important transcription factor of antioxidant genes, Forkhead box O3a (FOXO3a), in the lungs of COPD patients were similarly reported [100]. Of note, oxidative stress in COPD could further aggravate inflammation via several pathways such as the activation of NF-kβ, which may lead to the onset of depression in COPD.

**Table 2:** Shared mechanisms between COPD and mood disorders.

Mechanisms	References
Inflammation	
Elevated levels of inflammatory mediators such as CRP, fibrinogen, IL-6, IL-8, IL-13, MCP-1, TNF-α, and TNF-α receptor-1 were detected in the plasma of COPD patients.	[64,65,66,67,68,69]
Inflammatory cytokines such as CRP, IL-1, IL-6, IFN-γ, TNF-α, and TNF-α receptor 1 were associated with depressive symptoms in COPD.	[65,70,71,72,73]
Oxidative stress	
Decreased levels of reduced glutathione, as well as an increased amount of 8-isoprostane, were detected in COPD patients compared to controls.	[99]
Genetic polymorphisms associated with extracellular SOD as well as their expression in the sputum are frequently found in COPD patients.	[97,98]
Downregulation of Nrf2 and Nrf2-related, heme oxygenase-1, and glutamate-cysteine ligase catalytic subunit in the peripheral blood mononuclear cells were detected in COPD patients.	[99]
Reduced pulmonary expression of FOXO3a was reported in COPD patients.	[100]

COPD: Chronic obstructive pulmonary disease, CRP: C-reactive protein, FOXO: Fork-head box O, IL: Interleukin, IFN-γ: Interferon-γ, MCP: Monocyte chemoattractant protein, Nrf: Nuclear factor erythroid 2-related factor, TNF-α: Tumor necrosis factor-α, SOD: Superoxide dismutase.

A genome-wide association study (GWAS) reported common etiologies between depressive symptoms and COPD [101]. Among patients with COPD (n = 247) and controls (n = 119), GWAS showed a strong positive association between comorbid depression in COPD and a single-nucleotide polymorphism (SNP), rs3794808 in the SERT gene [102]. A study found no association between current anxiety and depressive symptoms in COPD patients (n = 302) with a polymorphism in the SERT gene in the promoter region (*5-HTTLPR*) and intron 2 variable number tandem repeat (*STin2VNTR*, 9, 10, or 12-repeat alleles); however, the polymorphism was associated with previous anxiety and depressive episodes [103]. The SERT gene is linked with CS via serotonin reuptake [104] and depression. *GC* (rs4588 and rs7041) and *VDR* (Bsm, Taql, Fokl) gene polymorphisms have been linked to exacerbation frequencies in COPD [105], especially in vitamin D-deficient COPD subjects [106,107,108]. Epigenetic changes resulting from CS also lead to increased mood disorders in COPD. In an animal study, CS enhanced the acetylation of H3K9 and modulated the expression of protease and pro-inflammatory genes via histone deacetylase (HDAC)-1 depression in rat lungs and macrophages [109]. In addition, CS is related to genomic alterations in DNA methylation in aryl hydrocarbon receptor repressor lung macrophages and lymphoblasts [110]. Glucocorticoid resistance also characterizes depression [111]. A study reported that inflammation in the central nervous system resulting from CS had induced glucocorticoid resistance in the rat model of COPD [112]. Moreover, polymorphism in 765G/C in the promoter of the cyclooxygenase (COX)-2 gene was linked with the advent of depression. On the other hand, the 765G/C polymorphism was not found to be associated with depression in COPD; in fact, it confers some resistance against COPD [113]. Prenatal activities could also be important factors in the predisposition to depression in COPD populations. For example, cytotoxicity during fetal development due to either bacterial or viral infections has been associated with a heightened predisposition to depression later in adult life [114]. Therefore, epigenetic changes resulting from CS, polymorphism in the SERT gene, and prenatal exposure to toxins could also increase the likelihood of mood disorders in COPD.

## 4. Potentials of n-3 PUFAs in Managing Comorbid Mood Disorders in COPD

n-3 PUFAs have reported benefits in diverse conditions such as cardiovascular disorders (CVD) [115,116], anxiety and depression [117,118,119,120], and inflammatory lung disorders, including asthma [121,122] and COPD [123,124,125]. A randomized controlled trial (RCT) showed a lowered incidence of IFN-α induced depression in patients with hepatitis C virus supplemented with EPA (10%) when compared with the placebo group (30%) prior to the IFN-α treatment [118]. Moreover, the manifestation of depression was significantly delayed by the EPA (12 weeks) and DHA (11.7 weeks) when compared with the placebo group (5.3 weeks) [118]. In addition, several meta-analyses reported a significant benefit of n-3 PUFAs supplementation on depression compared with a placebo [117,126,127]. A significant improvement was reported in the cognitive symptoms in CVD patients with comorbid Major Depressive Disorder (MDD) in week 8 during a 12-week RCT with n-3 PUFAs [120]. However, improvement in depressive symptoms was only seen in patients with comorbid severe MDD in CVD at the end of week 12 [120].

Increased intake of n-3 PUFAs or their increased plasma concentrations are linked to reduced anxiety, depression, and occurrence. An inverse correlation was observed between the consumption of n-3 PUFAs and the prevalence of depression among adolescent boys [128]. Similarly, fish consumption has been linked to reduced occurrence of major depression [129]. A significant association between increased plasma n-3 PUFAs concentration and reduced depressive symptoms in healthy adults was also reported [130]. Moreover, significantly lower ALA and total levels of n-3 PUFAs in serum cholesteryl esters and decreased EPA in phospholipid fractions and serum cholesteryl esters were seen in the MDD subjects [131]. Patients with severe to moderate depression reported a significant positive correlation between their AA to EPA ratio in tissues and depression severity [132]. A recent meta-analysis showed reduced depressive symptoms associated with low n-6/n-3 supplementation [133]. Increased n-3 PUFAs consumption results in increased grey matter in the brain regions, which is critical in regulating depression [134]. On the other hand, studies on the relationship between n-3 PUFAs and mood disorders in COPD are non-existent; hence, whether low erythrocyte or plasma n-3 PUFAs is associated with heightened mood disorders in this at-risk population is uncertain. Thus, future studies are needed in this regard.

**Figure 4:**
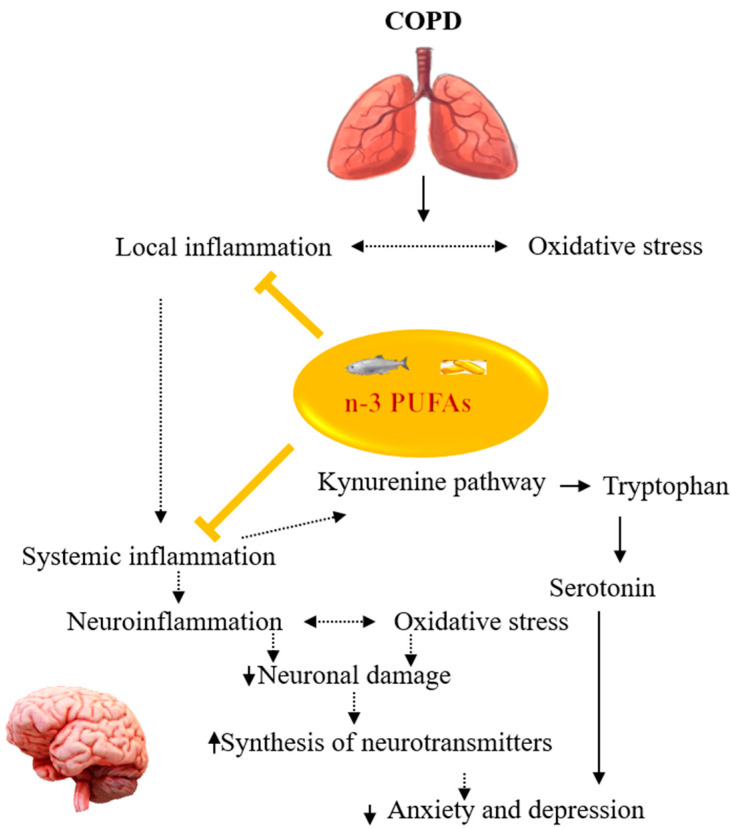
Summary of the potentials of n-3 PUFAs in managing COPD comorbid mood disorders. n-3 PUFAs can inhibit pulmonary and systemic inflammation thus reducing anxiety and depression in COPD. COPD: Chronic obstructive pulmonary disease, n-3 PUFAs: omega-3 polyunsaturated fatty acids.

Systemic inflammation has been suggested as the major link between COPD and comorbid anxiety and depression [135,136] (Figure 4). There is growing evidence that n-3 PUFAs can mitigate inflammation in COPD. For instance, a negative association between a higher intake of ALA and lower serum TNF-α in 250 stable COPD patients was reported [137]. In addition, a higher intake of AA was positively associated with higher serum IL-6 and CRP levels [137]. In another study, n-3 PUFAs supplementation combined with lycopene and rosuvastatin lowered the plasma IL-6 levels and reverted the leukotriene B4 receptor gene expression to baseline levels in COPD patients [124]. Meanwhile, reduced levels of IL-6, IL-8, and TNF-α in COPD patients with cachexia were reported after supplementation with personalized medical nutrition containing high-dose n-3 PUFAs combined with vitamin D and high-quality protein [125]. Sugawara et al. similarly reported reduced serum levels of hs-CRP, IL-6, IL-8, and TNF-α in COPD patients following supplementation with a nutrition drink containing n-3 PUFAs and vitamin A as an adjunct to low-intensity exercise [138]. Moreover, a meta-analysis reported reduced IL-6 in patients with COPD after supplementation with n-3 PUFAs compared to placebo [139]. n-3 PUFAs have been demonstrated to suppress pro-inflammatory mediators production, reduce the transcription of cell adhesion elements on monocytes and endothelial cells, and suppress the formation of free radicals by neutrophils [140]. Moreover, n-3 PUFAs may also block the NF-kβ [32], limiting the production of pro-inflammatory cytokines. Furthermore, n-3 PUFAs prevent the synthesis of pro-inflammatory lipid mediators such as prostaglandin E2 and leukotriene B4 by inhibiting COX-2 activity or antagonizing the formation of AA from membrane phospholipids [62,141]. In addition, the suppression of the production of pro-inflammatory lipid mediators and the inhibition of COX-2 also suppress the activity of IDO enzymes involved in the Kyn pathway; hence less TRP is degraded into kynurenines [142], and this ensures the plasma availability of the TRP that will be transported to the brain for serotonin synthesis. Apart from their anti-inflammatory action, EPA and DHA may be beneficial in depression through their effects on neuroplasticity [143,144,145], an important molecular mechanism for the actions of antidepressants [146,147,148]. Metabolites of n-3 PUFAs, specialized pro-resolvin mediators, including RvD and E, protectins, and maresins (MaR) also mediate inflammatory pathways via their resolution potentials [28,149]. n-3 PUFAs may also be beneficial for the resolution of bronchial inflammation associated with COPD. Indeed, significantly lower free ALA and EPA were detected in the sputum of stable COPD patients compared with normal controls [150]. Moreover, higher levels of AA were reported in the acute exacerbation of COPD compared to the stable phase [150]. Furthermore, an in vivo study reported a reduced bronchoalveolar lavage neutrophil infiltration of IL-6, TNF-α bronchiolar inflammation as a result of acute and recurrent exposures to organic dust following treatment with MaR1 [150]. In addition, MaR1 dose-dependently reduced the IL-6 and IL-8 production in the organic dust-exposed bronchial epithelial cells [151]. In sum, n-3 PUFAs or their metabolites could target both systemic and bronchial inflammation associated with COPD, thereby reducing the onset of COPD-related mood disorders.

n-3 PUFAs modulate the antioxidant system. Supplementation with n-3 PUFAs has reduced the oxidative stress, indicated by elevated levels of 8-isoprostane, advanced oxidation protein products, nitrotyrosine, and increased Trolox equivalent antioxidant capacity and SOD activity in pediatrics and adolescents with depressive symptoms compared with the healthy control group [152]. Similarly, an inverse relationship was reported between erythrocyte n-3 index and depressive symptoms in subjects with high oxidative stress biomarkers [153]. In addition, a negative correlation was reported between the iNOS, thiobarbituric acid reactive substances, nitrotyrosine and erythrocyte n-3 PUFAs [91]. Moreover, a significant decrease in malondialdehyde level with a concurrent reduction in depressive symptoms was observed in patients treated with n-3 PUFAs who were depressed at baseline [154]. n-3 PUFAs have also been reported to improve rat antioxidant defense in astrocytes via the Nrf2-dependent mechanism [155]. Similarly, the upstream pathways of Nrf2 depend on the ratio of DHA/EPA incorporated into the membrane phospholipids [155]. 

The evidence above suggests that n-3 PUFAs could reduce the incidence of mood disorders in COPD by mitigating inflammation and oxidative stress, which are shared mechanisms between mood disorders and COPD. However, studies in this area are very limited as only one study examined the effect of personalized nutrition therapy (containing n-3 PUFAs) as an addition to exercise training (ET) in COPD in a controlled trial [156] with mood disorders as exploratory outcomes [156]. In the study, muscle-wasted COPD patients enrolled in an outpatient pulmonary rehabilitation program received either a supplement fortified with leucine, vitamin D and n-3 PUFAs or a placebo [156]. The supplement group showed a substantial reduction in anxiety and depressive symptoms based on the Hospital Anxiety and Depression Scale (HADS) compared to the placebo group [156]. However, because the trial included multi-nutrient supplementation in addition to ET in COPD patients, the improvement in HADS score may not be attributable to n-3 PUFAs alone. Thus, future studies with mood disorders as main outcomes are needed to see whether supplementation with n-3 PUFAs will be beneficial in reducing the incidence of mood disorders in the COPD population.

## 5. Conclusions

Mood disorders are important comorbidities in COPD with varying negative effects on treatment adherence, QOL, and the development of COPD progression. Despite their negative impacts on COPD clinical outcomes, mood disorders are often neglected or go undetected in the clinical management of the disease. Inflammation, oxidative stress, and hypoxia are fundamental mechanisms leading to an increased prevalence of anxiety and depression in COPD patients. Pharmacological therapies are frequently used in managing mood disorders in COPD; however, as their use is associated with multiple side effects, patient adherence is limited. On the other hand, n-3 PUFAs (including their metabolites) may be a potential treatment to help manage anxiety and depression in COPD patients due to their ability to modulate inflammatory pathways and activate antioxidant defense systems. However, studies on the effect of n-3 PUFAs in COPD comorbid mood disorders are very limited. Therefore, studies are warranted to assess the relationship between n-3 PUFAs and comorbid mood disorders in COPD and evaluate the effect of n-3 PUFAs supplementation on comorbid mood disorders in COPD.

## Figures and Tables

**Table 1:** Important Sources of n-3 and n-6 PUFAs.

Fatty Acid	Important Sources
ALA	flaxseed, canola, and soybean
EPA	Fish and fish oils
DHA	Fish oil and brown algae
LA	Corn, safflower, sunflower
ALA	Dairy products, eggs, and meats

ALA: Alpha-linolenic acid, EPA: Eicosapentaenoic acid, DHA: Docosahexaenoic acid, LA: Linoleic acid; AA, Arachidonic acid.

## Data Availability

Not applicable.
